# Capacitive Sensing of Icing under Vacuum and Cryogenic Temperatures

**DOI:** 10.3390/s19163574

**Published:** 2019-08-16

**Authors:** Juliana Padilha Leitzke, Tobias Mitterer, Hubert Zangl

**Affiliations:** Institute of Smart Systems Technologies, Sensors and Actuators, Alpen-Adria-Universität Klagenfurt, Klagenfurt 9020, Austria

**Keywords:** capacitive sensors, icing, ice formation

## Abstract

In certain industrial processes, ice aggregations on surfaces can occur under almost vacuum conditions and at very low to cryogenic temperatures due to residual water molecules. This aggregation can affect the performance of the process and it is therefore of interest to monitor such surfaces. In this paper, we present a capacitive ice measurement system capable to operate in vacuum and temperatures of about −120 ${}^{\circ }C$ and below. We present a capacitive sensor setup with a separation of sensor element and sensor electronics, such that the sensor electronics can reside outside the cold environment. It is demonstrated that the permittivity of such ice formations at vacuum and low temperatures is sufficient for measurement using the proposed sensor configuration. Results from a long-term study using a prototype further demonstrate the stability of the system and thus the feasibility of the proposed system for long term condition monitoring of surfaces in vacuum that are e.g., cooled by cryogenic liquids. The developed system uses wireless communication in order to allow for simple retrofitting of existing infrastructure even in remote locations.

## 1. Introduction

Ice formation can be present in many applications and can adversely affect the performance of a system. Examples are heat exchangers in cooling systems, ice aggregation on wind turbines or overhead power-lines or even icing on surfaces of aircraft. Capacitive sensors have been considered a reliable method for ice detection in such applications [1,2,3,4], also with respect to detection of ice and water mixtures [5]. In [6], the use of wireless sensors for water ice probing on the moon is proposed and a sensor based on the measurement of electrical properties like a capacitive sensor has been suggested as one of the possible sensing methods for that system. Furthermore, in certain industrial applications, ice can occur under almost vacuum conditions and very low temperature, e.g., on surfaces cooled by cryogenic liquids such as liquid nitrogen, and monitoring this ice formation can improve the operation of the system.

## 2. Background

The permittivity of ice varies according to frequency and temperature [7], as well as environmental conditions such as pressure, which affect the way ice is formed [8]. The behavior of ice properties in frequency have been well-studied in the literature [9,10] and a commonly used model of the complex permittivity of ice considering a constant temperature is given by

(1) $$\varepsilon =\varepsilon^{\prime }-j\varepsilon^{\prime \prime }$$

(2) $$\varepsilon^{\prime }=\varepsilon_{\infty }+\frac{\left(\varepsilon_{s}-\varepsilon_{\infty }\right)}{1+\omega^{2}\tau_{0}^{2}}$$

(3) $$\varepsilon^{\prime \prime }=\frac{\omega \tau_{0}\left(\varepsilon_{s}-\varepsilon_{\infty }\right)}{1+\omega^{2}\tau_{0}^{2}}$$

where ε is the complex permittivity, $\varepsilon^{\prime }$ is the real permittivity, $\varepsilon^{\prime \prime }$ is the imaginary permittivity, $\varepsilon_{\infty }$ is the permittivity in high frequency, $\varepsilon_{s}$ is the static permittivity, ω is the angular frequency, and $\tau_{0}$ is the relaxation time.

**Figure 1:**
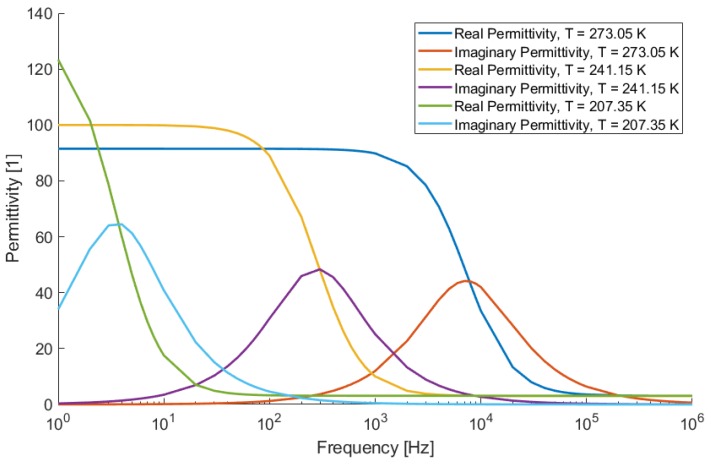
Permittivity dependency on frequency and temperature [11].

In [11], the complex permittivity of ice was measured from melting point down to 208.15 K and, while the relaxation time and static permittivity were found to be highly dependent on temperature, the high frequency permittivity is fairly constant, which is exemplified in Figure 1. As it can be extrapolated that the cut-off frequency at lower temperatures will be quite low (far below 10 Hertz) making accurate measurements can be difficult. Nevertheless, the higher frequency permittivity still significantly differs from the permittivity of vacuum but type and density of ice may still affect the actual effective permittivity.

Ice can be formed in many different ways, hexagonal ice, or ice lh, is the most common form of ice which is formed under normal pressure conditions on Earth. In [12], the high frequency permittivity of hexagonal ice is investigated to temperatures as low as 2 K, being also modelled depending on temperature as

(4) $$\varepsilon_{\infty }=3.093\pm 0.003+\left(0.72\pm 0.60\right)10^{-4}T+\left(0.11\pm 0.02\right)10^{-5}T^{2}$$

where *T* is the temperature. The value of the high frequency permittivity varied between 3.093 ± 0.003 at 2 K to 3.15 ± 0.02 at 200 K.

The high frequency permittivity of crystalline ice was more recently addressed in [13,14], the stable value obtained for 253.15 K was 3.16±0.01. There it is shown that as the density of the ice is reduced, as for example due to air bubbles, the permittivity is also reduced.

According to [15], the expected conditions in the targeted applications will lead to cubic crystalline ice, Ic, formed from vapor deposition. Water is not present in the liquid state under low pressure and low temperature and ice formation under such conditions and its sublimation is also addressed by [16].

Due to the icing conditions, the density and thus the permittivity of the ice can be quite low under such conditions as shown in [14]. In case we have ice as host medium with empty inclusions of volume fraction *f*, the resultant permittivity could be given by the model in Equation (5) [17], where $\varepsilon_{v}$ is the permittivity of vacuum and $\varepsilon_{i}$ is the permittivity of ice.

(5) $$\varepsilon =\varepsilon_{i}\frac{\varepsilon_{i}+\frac{1+2f}{3}\left(\varepsilon_{v}-\varepsilon_{i}\right)}{\varepsilon_{i}+\frac{1-f}{3}\left(\varepsilon_{v}-\varepsilon_{i}\right)}$$

**Figure 2:**
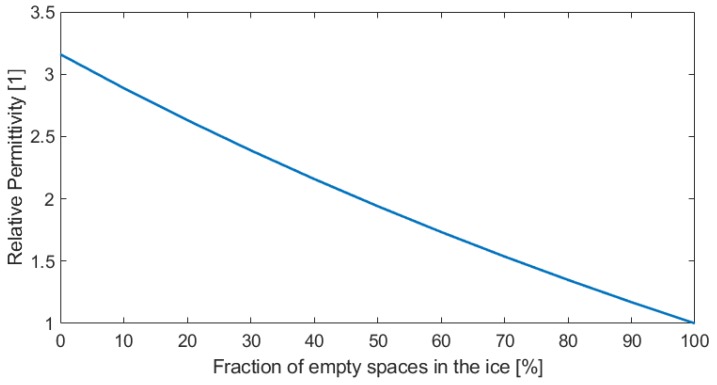
Permittivity behavior for empty inclusions in the ice.

This equation is exemplified in Figure 2, where the resultant permittivity value for different fractions of empty spaces in the ice is shown.

Consequently, a sensor for ice aggregation in the described application scenarios is feasible, provided that the density of the formed ice is high enough under the given conditions, which was further investigated experimentally.

## 3. Methods

The feasibility was initially assessed through simulations and climate chamber tests of the prototype in the laboratory. As a last step the prototype was installed in the equipment under vacuum and low temperatures and data was collected over several months for analysis.

### 3.1. Simulations

**Figure 3:**
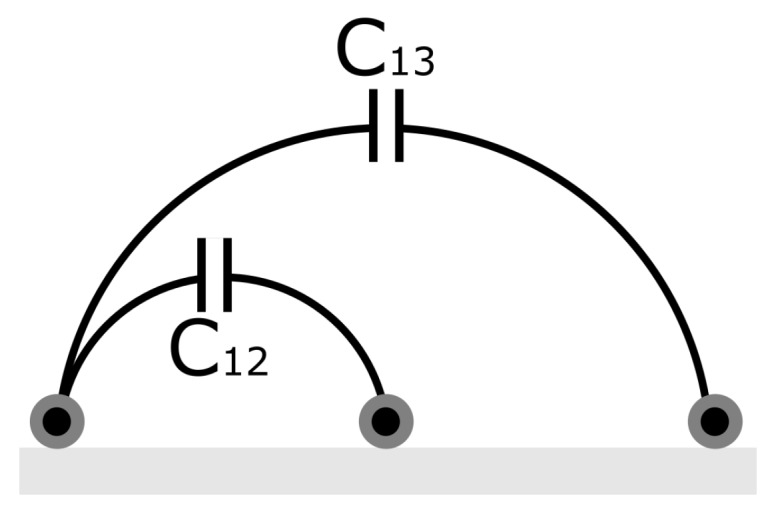
Capacitance between electrodes in differential measurement mode.

FEM (Finite Element Method) simulations were made to determine an appropriate length for the electrodes. The electrodes were assumed to be formed by the two inner layers of an in-vacuum coaxial cable consisting of an inner conductor layer and an outer insulating layer, where the conductor has 0.61 mm of diameter. The geometry used for simulations consisted of three electrodes placed at a fixed distance as shown in Figure 3 and a material placed on them that changed its thickness from 1 mm until 1 cm. Such a geometry was chosen since it was already proposed and verified in [1] for overhead power lines. From this previous work, it is known that the capacitance between electrodes goes into saturation at an ice thickness that is related to the distance between the electrodes. Therefore, small distances provide a larger signal, but that is valid only for thin layers. Additionally, the permittivity of the ice created under these conditions was not known and assumed to be very low. Using simulations, we decided on a design that would allow for the measurement of ice layers into the range of several millimeters even at very low permittivity.

**Figure 4:**
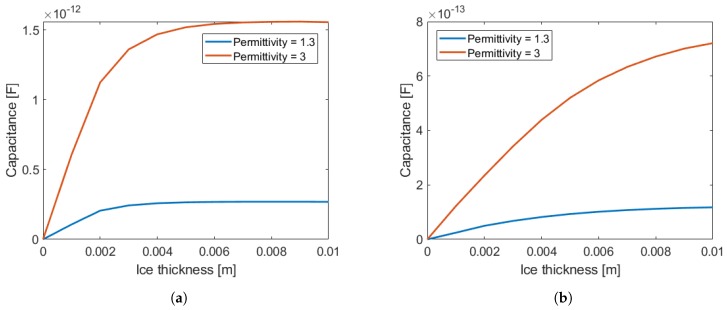
Simulation results for a permittivity of 1.3 and 3. In (**a**), the capacitance between the first and second electrode in differential measurement mode is shown, while in (**b**) we have the capacitance between the first and third electrode.

In Figure 4a,b below, results are shown for the obtained capacitance values considering differential mode and an electrode length of 20 cm. In Figure 4a, capacitance values are obtained on the first electrode when the signal is received from the second electrode, while in Figure 4b when the signal is generated on the third electrode. In these figures, the capacitance for a permittivity of 1.3 and 3 are shown, already considering the difference to a vacuum reference. These values were chosen because ice has a permittivity of approximately 3 at higher frequencies, but it is possible that this permittivity gets closer to 1 under these conditions. In these images, we can see that the capacitance value varies according to the thickness of the material.

It is also wished that the capacitance value stays within the operation range to be used in the proposed hardware and that the values vary according to the resolution of our hardware. This is taken into consideration when choosing the appropriate electrode length.

### 3.2. Measurement System

**Figure 5:**
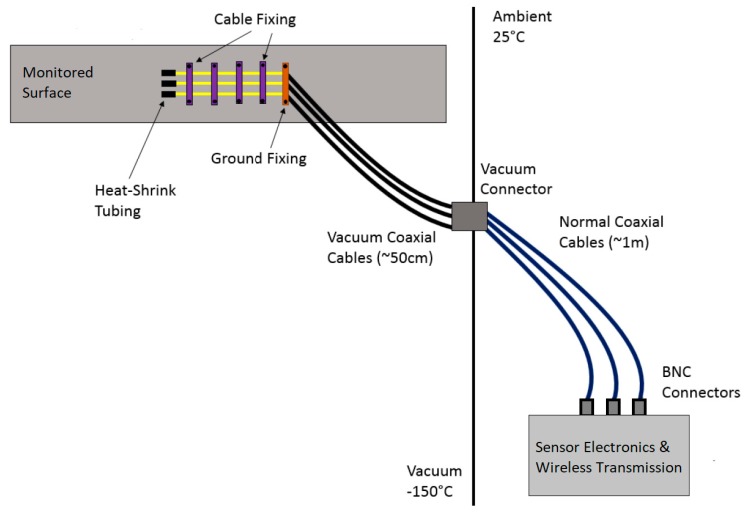
Overview of the proposed measurement system.

Based on the discussion in the previous sections, a frequency of 250 kHz was chosen for the capacitive sensor system which is expected to be significantly above the cut-off frequency and thus in a range where the permittivity is less temperature dependent. In the proposed system (compare Figure 5), the sensor electrodes are separated from the sensor electronics, such that the electronics can reside outside of the vacuum chamber. This brings the additional advantage that wireless communication can be used, which would be prohibited by the vacuum chamber as it is usually made of metal. Using the differential sensing mode, the influence of cable capacitances is negligible, as discussed e.g., in [18].

The capacitive sensor was created using in-vacuum coaxial cables and the ground shield was the equipment where it was fixed. These electrodes were connected to an AD7142, a Capacitance-to-Digital Converter (CDC) with a resolution better than 1 fF, that transmitted the values via I2C to a nRF51822 micro-controller, which sent the samples of the two connected electrodes to a base-station via a wireless connection using the 2.4 GHz band with a fixed transmission rate.

The base station was implemented using a Beaglebone Black Wireless (BBBW), which is a low-cost development platform. The software has been implemented in python for the base-station functionality, where the stability and reliability is ensured by a software supervisor, which checks continuously if the main script is still running and which restarts the system otherwise.

The base-station functionality comprises the communication with the sensor where measured samples are being transmitted continuously. The sensor node is connected via wireless and not via cable to enable the sensor node to be mounted more flexibly if the need arises. On the base-station all received samples are supplied with time-stamps and stored in a buffer and in files in the local memory. For monitoring, supervision and cloud backup of the system, an Universal Serial Bus (USB) 4G modem is used.

**Figure 6:**
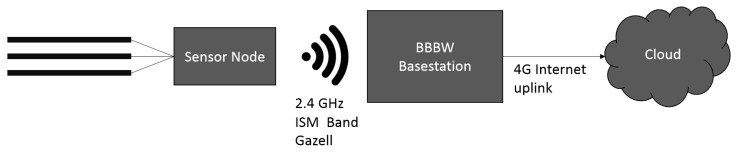
Diagram depicting the measurement system.

**Figure 7:**
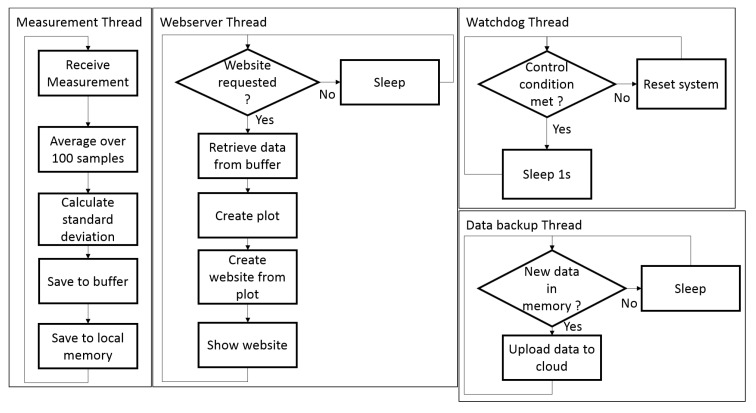
Flowgraph depicting the internal structure of the measurement system.

For this test, the sensor and the BBBW were mounted in the same closed package as the there was enough space for both systems. To monitor the hardware as well and record the influence of the temperature of the electronics on the measurement, an additional temperature and humidity sensor was added and connected via I2C directly to the BBBW. The setup is displayed in in Figure 6 and the internal process of the system is displayed in Figure 7 as a flow graph.

### 3.3. Laboratory Experiments

#### 3.3.1. Temperature Dependence

**Figure 8:**
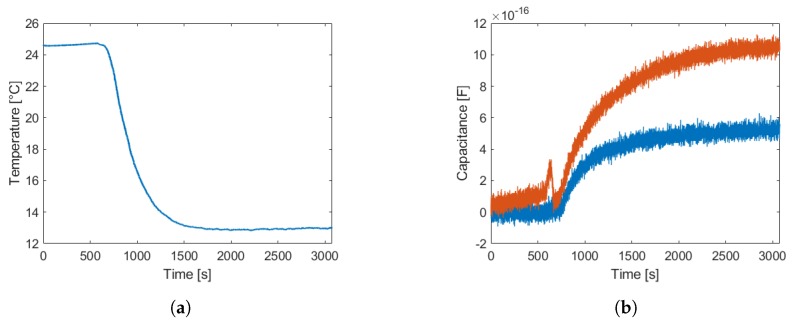
Temperature sensitivity experiments. In (**a**) the temperature variation to which the sensor was exposed to is shown and in (**b**) the measured capacitance difference value.

The sensitivity needs to be compared to cross-sensitivities, in particular towards temperature. This applies to both, the sensor electronics and the sensor front end. The cross-sensitivity towards temperature variations is studied in the climate chamber and results are shown in Figure 8 for a temperature variation of approximately 12 K. It is possible to see that the capacitance variation is very low.

The electronic circuitry will be placed outside of the vacuum chamber, in a temperature-controlled environment. Therefore, the temperature variation in this environment is quite low. Furthermore, the sensor front end will be kept at fairly constant temperature inside the chamber and this cross-sensitivity can be considered of low relevance.

#### 3.3.2. Icing Experiment

**Figure 9:**
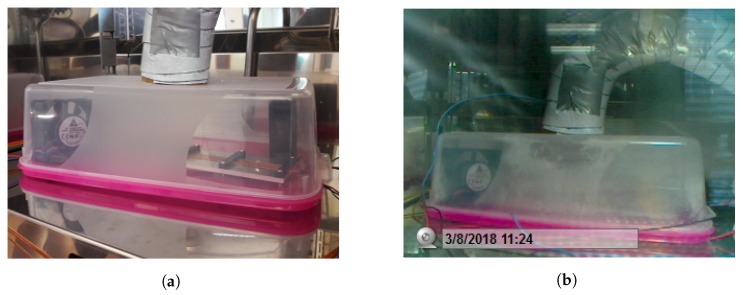
In (**a**), the laboratory setup for icing experiments is shown. The enclosed box is placed in the climate chamber and kept at low temperature. Ambient air with a dew point above the temperature in the chamber is blown in and the moister freezes on the cold surfaces. The hose for the air needs to be insulated in order to avoid freezing inside the hose. Fans inside the chamber are used to distribute the air (and thus the moisture) inside the box. In (**b**), an image from webcam during icing experiment is presented. Ice accretion on the surfaces of the sensor and the box can be observed.

In order to simulate ice accumulation on the sensor surface in the laboratory, a test setup according to Figure 9a was created. An example image as recorded during the experiment is shown in Figure 9b. During this experiment the sensor was placed inside the plastic box, in order to keep the humidity contained and avoid icing on the climate chamber structures. Humidity was pushed from outside into the box by means of a fan, inside the box two other fans were placed to circulate the air and distribute the icing.

**Figure 10:**
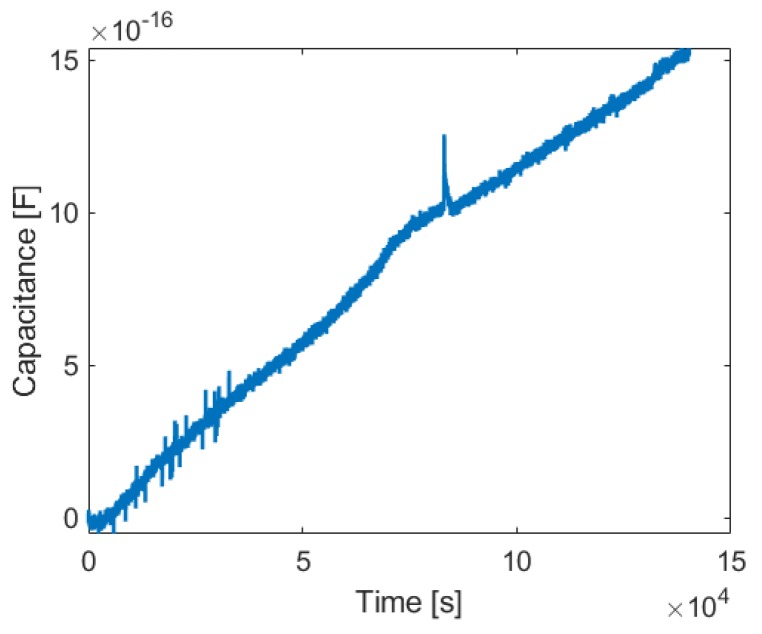
Experimental results for ice accretion on the sensor surface in the climate chamber experiment. The capacitance difference in one of the electrodes caused by the ice accretion at constant temperature is shown.

**Figure 11:**
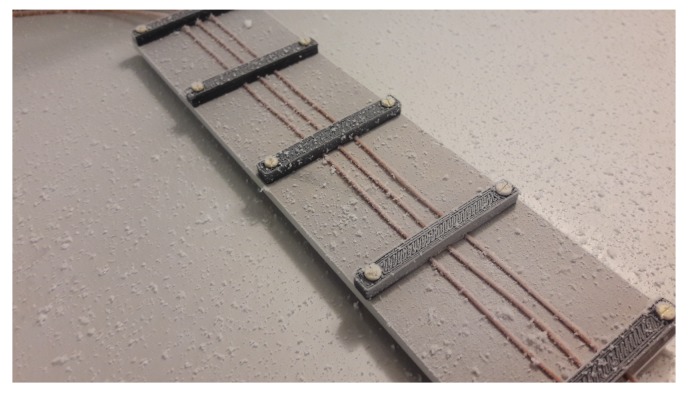
Example of ice layer formed during one of the climate chamber experiments. Here it is possible to see the irregular way ice is formed in a sample with thickness varying from a few μm to a mm.

Results are shown in Figure 10 and an increasing capacitance can be observed despite the very fragile low density ice structures that are formed in the chamber as what is shown in Figure 11. Consequently, it is assumed that the system can also measure ice accretion with low permittivity values at even lower temperature.

## 4. Discussion and Results

**Figure 12:**
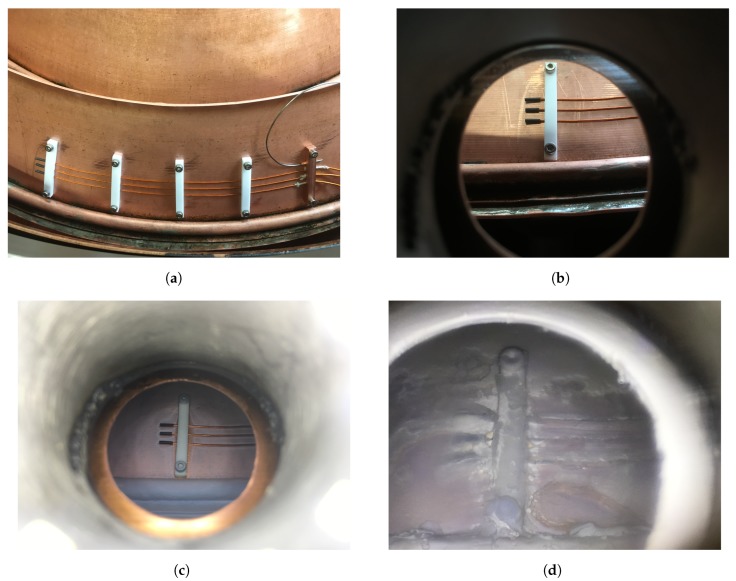
Electrodes under long-term testing. In (**a**) the electrodes installed inside the equipment are shown, in (**b**) the window for visualization, and in (**c**,**d**) different icing states.

For the long-term experiments inside the equipment in the field, the electrodes were fixed as shown in Figure 12a. The cables were accessed by the measurement system from outside through an specific vacuum connector. In addition, a window for icing visualization was included as shown in Figure 12b. Different icing states are shown in Figure 12c,d.

**Figure 13:**
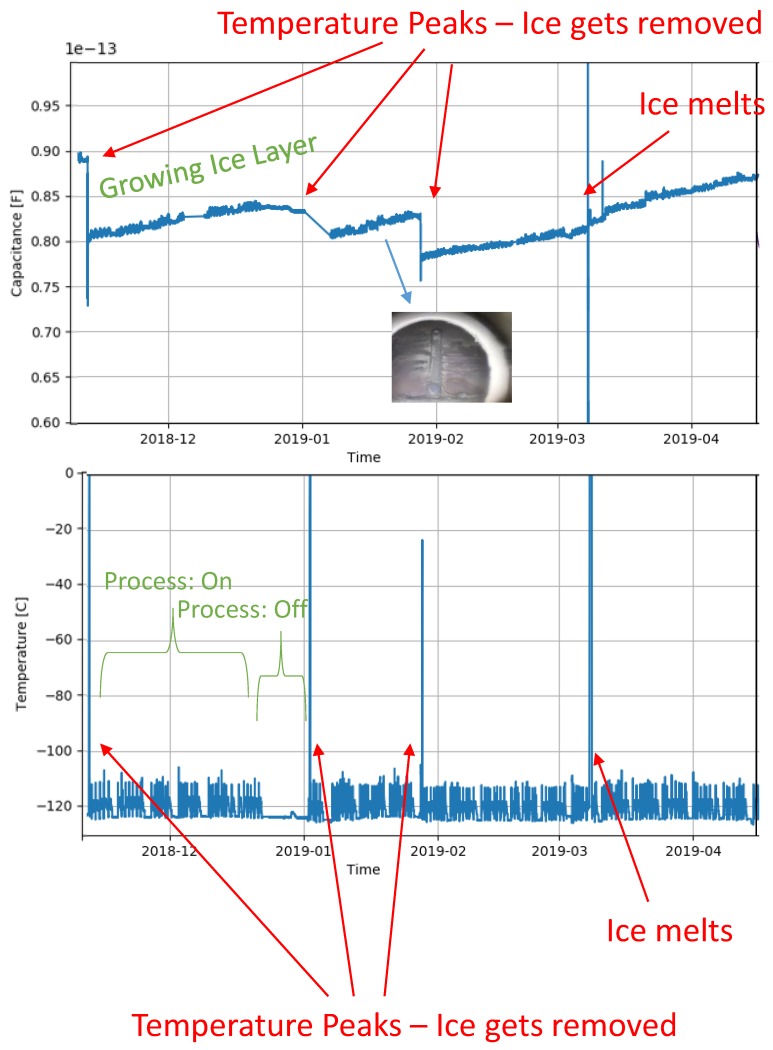
Capacitance and temperature measurement results over a long period of time showing the ice growth and also de-icing, which can be identified by the temperature peaks.

The temperatures inside the equipment were around 153.15 K and data was collected over several months. In Figure 13 the capacitance is shown for growing layers of ice. The temperature was monitored by a parallel system during part of the measurements and it can also be seen in Figure 13, where the larger peaks represent a de-icing process, where temperatures are increased and the presence of water is possible, which can change the offset conditions of the sensor.

The signals show some cross-sensitivity to the process probably due to some temperature increase inside the equipment and the variations of the industrial process itself, which cause the smaller peaks in the signal. This is possible to see when comparing the capacitance measurements to the process temperature plot.

**Figure 14:**
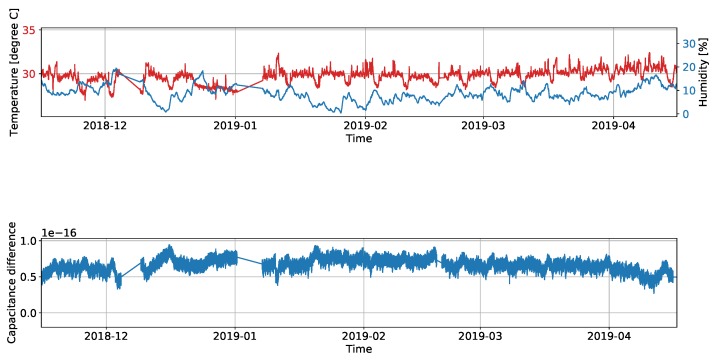
Temperature and humidity inside the electronics housing and reference capacitance difference measurement results of not connected channel of the sensor.

To further show the long-term stability of the measurement system, Figure 14 illustrates the measurements received from one of the electrode channels which is not connected to an electrode. The second plot shows that the capacitance change due to variations in the environment around the electronics is negligible. To further state that the measurements are not significantly dependant on the humidity or temperature around the electronic parts, the first plot in the figure displays the measurements of an humidity sensor and a temperature sensor placed inside the electronics housing, which were installed to check for dependencies of the measurements on the environmental influences around the electronics.

## 5. Conclusions

The study shows that capacitive ice measurement at the given conditions is feasible. The sensor front end and the measurement hardware worked continuously during the time of the experiment, short term data losses occurred due to non hardware-related issues such as an unplugged power connection in the field. Consequently, a long-term operation of such a system under the given environmental conditions is also feasible.

Even though the feasibility of the measurement system could be demonstrated, the threshold levels for the ice layer thickness have not been found as the degree of ice that occurred in the experiment was lower than expected. A change of the sensor front end may be required to optimally adjust the sensor for the required thickness. When the ice gets removed during periods of higher temperatures, water drops may remain on the sensor surface that lead to a change of the offset signal. Consequently, the offsets should be corrected every time the process restarts. As only three such events occurred during the test, the significance of the results should be enhanced by a continuation of the investigations.

## Abbreviations

The following abbreviations are used in this manuscript:

BBBW	Beaglebone Black Wireless
CDC	Capacitance-to-Digital Converter
FEM	Finite Element Method
USB	Universal Serial Bus
